# Trabekuläre Knochendichtemessung in Hounsfield-Einheiten im proximalen Femur zur Osteoporoseabklärung und Frakturrisikobestimmung

**DOI:** 10.1007/s00132-025-04726-4

**Published:** 2025-10-02

**Authors:** Julian Ramin Andresen, Thomas Haider, Reimer Andresen

**Affiliations:** 1 Klinische Abteilung für Unfallchirurgie, Universitätsklinik für Orthopädie und Unfallchirurgie, Medizinische Universität Wien, Währinger Gürtel 18–20, 1090 Wien, Österreich; 2 Institut für Diagnostische und Interventionelle Radiologie/Neuroradiologie, Westküstenklinikum Heide, Akademisches Lehrkrankenhaus der Universitäten Kiel, Lübeck und Hamburg, Heide, Deutschland

**Keywords:** Knochenverlust, altersbedingter, Knochenmineralgehalt, Femurkopf, Hüftfrakturen, Insuffizienzfrakturen, Bone loss, age-related, Bone mineral density, Femur head, Hip fractures, Insufficiency fractures

## Abstract

**Hintergrund:**

Osteoporose führt zu Frakturen, insbesondere bei älteren Patienten nach Bagatelltraumata. Häufig kommt es zu Hüftfrakturen.

**Fragestellung:**

Untersucht wurde, ob sich anhand der trabekulären Dichte in Hounsfield-Einheiten (HU) im proximalen Femur Knochendichte (KMG) und T‑Scores berechnen sowie das Vorliegen einer Osteoporose und das Frakturrisiko abschätzen lassen.

**Methode:**

370 Patienten (Ø 69,4 Jahre, 53 Männer/317 Frauen) wurden mittels CTXA-Hüfte (DEXA-Äquivalent) untersucht. Zusätzlich erfolgte die HU-Messung im CT-Schnittbild im Caput femoris und proximalen Femur. Frakturen wurden durch ergänzende Bildgebung erfasst.

**Ergebnisse:**

Mit zunehmendem Alter und abnehmendem BMI nahm die trabekuläre Knochendichte signifikant ab. Es bestand eine starke Korrelation zwischen HU-Werten und KMG (R^2^ = 0,88) bzw. T‑Score (R^2^ = 0,89). Ein Schwellenwert von 96 HU zeigte eine hohe Vorhersagekraft für Osteoporose (AUC = 0,97) für den Bereich des proximalen Femurs, für das Caput femoris lag der Schwellenwert bei 245,5 HU. Der Frakturschwellenwert lag für das Caput femoris bei 245,5 HU und für das proximale Femur bei 75 HU.

**Diskussion:**

Die HU-Messung im nativen CT-Schnittbild erlaubt eine verlässliche Abschätzung von Osteoporose und Frakturrisiko. Eine opportunistische Auswertung ist praktikabel und zuverlässig möglich.

**Graphic abstract:**

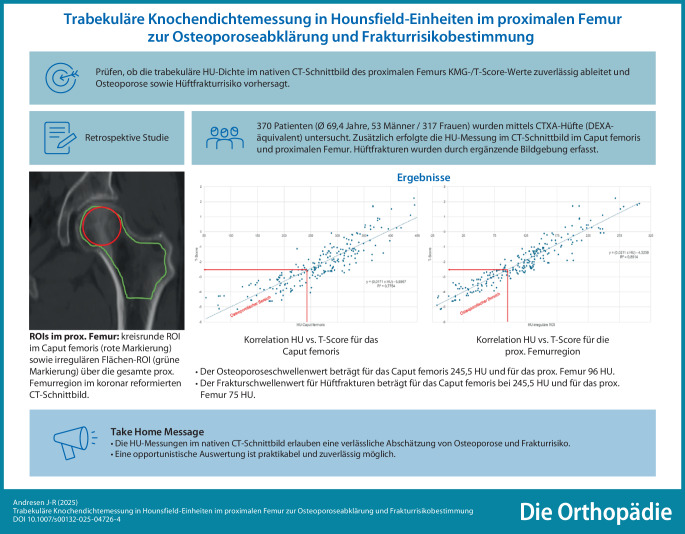

## Abstract (en)

**Background:**

Osteoporosis leads to fractures, especially in older patients after minor trauma. Hip fractures are frequent.

**Research question:**

The study investigated whether bone mineral density (BMD) and T‑scores can be calculated based on trabecular density in Hounsfield units (HU) in the proximal femur, and whether the presence of osteoporosis and fracture risk can be assessed.

**Patients and methods:**

370 patients (Ø 69.4 years, 53 men/317 women) were examined using CTXA hip (DEXA equivalent). In addition, HU measurements were taken in the CT cross-section of the caput femoris and proximal femur. Fractures were recorded using supplementary imaging.

**Results:**

Trabecular bone density decreased significantly with increasing age and decreasing BMI. There was a strong correlation between HU values and BMD (R^2^ = 0.88) as well as T‑score (R^2^ = 0.89). A threshold value of 96 HU showed high predictive power for osteoporosis (AUC = 0.97) for the proximal femur region, for the femoral head, the threshold value was 245.5 HU. The fracture threshold value was 245.5 HU for the femoral head and 75 HU for the proximal femur.

**Discussion:**

HU measurement in native CT cross-sectional images allows a reliable assessment of osteoporosis and fracture risk. Opportunistic HU evaluation alone is, therefore, practicable and sufficient.

Am häufigsten kommt es zu Frakturen der Hüfte bei älteren Patienten nach einem Bagatelltrauma. Meist ist ein erhöhtes Frakturrisiko aufgrund einer vorhandenen Osteoporose nicht bekannt und somit liegt auch keine antiosteoporotische medikamentöse Therapie vor. Aus unterschiedlichen medizinischen Indikationen liegen häufig CT-Untersuchungen der Beckenregion vor, hier könnte die Bestimmung der trabekulären Knochendichte in Hounsfield-Einheiten (HU) im proximalen Femur eine Abschätzung einer vorliegenden Osteopenie/Osteoporose ohne zusätzliche Strahlenbelastung ermöglichen.

## Einleitung

Ein fortschreitender Verlust an Knochenmineralgehalt (KMG) führt zur Entwicklung einer Osteoporose, die mit einem erhöhten Frakturrisiko sowohl am Achsenskelett [1] als auch in peripheren Bereichen einhergeht. Besonders häufig treten Frakturen der Hüfte, des distalen Radius und des prox. Humerus auf [24, 27]. Bei Patienten mit Hüftfrakturen finden sich zudem in großer Zahl unbemerkte Sinterungsfrakturen der Wirbelsäule [27]. Langfristig führen diese Frakturen zu einer erheblichen Einschränkung der Lebensqualität und einer erhöhten Mortalität [10, 39].

Die Zahl der Hüftfrakturen lag 1990 weltweit bei etwa 1,7 Mio. und wird angesichts des demografischen Wandels für das Jahr 2050 in einer Studie aus dem Jahr 1992 auf rund 6,3 Mio. geschätzt [10]. Dieses wurde durch eine neuere Schätzung bereits für das Jahr 2019 mit weltweit 14,2 Mio. registrierten Hüftfrakturen bei weitem übertroffen [16]. Eine Analyse der Krankenhausdiagnosestatistik für Deutschland aus dem Jahr 2004 ergab, dass jährlich rund 116.000 Personen eine Hüftfraktur erleiden [22]. Frakturen des Schenkelhalses und der pertrochantären Region zeigten in Deutschland von 2009 bis 2019 einen Anstieg von mehr als 20 %. Für 2019 betrug die Inzidenz von Schenkelhalsfrakturen 120,2 pro 100.000 und die von pertrochantären Frakturen 108,7 pro 100.000 Einwohner [35], während die Zahl der operativen Eingriffe derzeit bei etwa 135.000 pro Jahr liegt. Hüftfrakturen stellen nicht nur eine medizinische, sondern auch eine erhebliche sozioökonomische Herausforderung dar, die Behandlungskosten belaufen sich für Deutschland auf etwa 2,8 Mrd. € pro Jahr und werden vermutlich aufgrund der zunehmenden Alterung auf circa 3,85 Mrd. € bis 2030 ansteigen [43].

Für Patienten mit osteoporotischen Hüftfrakturen beträgt die kumulative Mortalitätsrate im ersten Jahr nach dem Frakturereignis 20–40 % [7, 26]. Eine spanische Studie zeigt, dass Männer mit einer Mortalitätsrate von 43 % im ersten Jahr nach der Fraktur ein höheres Sterberisiko haben als Frauen mit 30 % [20]. Zudem erreichen lediglich 30–40 % der Betroffenen wieder ihr ursprüngliches Mobilitätsniveau [12]. Patienten, die nach einer Hüftfraktur pflegebedürftig werden, profitieren von einer orthogeriatrischen Versorgung, die sich positiv auf funktionelle Ergebnisse, die Reduktion von Folgefrakturen und die Mortalitätsrate auswirkt [19, 21].

Zu den wesentlichen Risikofaktoren für Osteoporose sowie für das Auftreten von hüftgelenksnahen Frakturen zählen ein hohes Alter, ein niedriger KMG, Diabetes mellitus Typ I und II, vorangegangene Frakturen (Hüfte, Becken, Wirbelsäule, Humerus), Morbus Parkinson, Morbus Alzheimer, Multiple Sklerose, Epilepsie, eine langfristige Glukokortikoidtherapie, rheumatoide Arthritis, ein niedriger BMI, Vitamin-D-Mangel, weibliches Geschlecht, Hyponatriämie sowie Nikotin- und Alkoholkonsum [4, 25, 45, 48]. Über 90 % der Hüftfrakturen bei älteren Menschen entstehen direkt infolge eines Sturzes aus geringer Höhe, hierbei zeigen insbesondere Frauen im hohen Alter einen signifikanten Anstieg von intertrochantären Frakturen [13].

Als Goldstandard zur Bestimmung der Knochendichte und Diagnose einer Osteoporose gilt die Dual-Energie-Röntgenabsorptiometrie (DEXA) [15]. Ein T‑Wert zwischen −1,5 und −2,5 definiert eine Osteopenie, während Werte niedriger −2,5 als Osteoporose klassifiziert werden [14]. Als alternative Methode ermöglicht die CTXA-Hüfte ebenfalls eine zuverlässige Ermittlung DEXA-äquivalenter Werte in mg/cm^2^ und T‑Werten, wodurch sich eine Osteoporose in diesem Bereich gut diagnostizieren lässt [6].

Diese Studie untersucht, inwieweit sich das Ausmaß einer Osteopenie/Osteoporose und das Frakturrisiko an der Hüfte anhand der trabekulären Dichtebestimmung in Hounsfield-Einheiten (HU) im nativen CT-Schnittbild des prox. Femurs abschätzen lassen. Zudem soll überprüft werden, ob sich aus den HU-Werten quantitative KMG- und T‑Werte berechnen lassen und wie diese im Vergleich zu den CTXA-Werten einzuordnen sind.

## Patienten und Methode

### Studiendesign und Ethikvotum

Es handelt sich um ein retrospektiv untersuchtes Patientenkollektiv, für das ein Ethikvotum der zuständigen Universitätsmedizin (AZ: D 471/24, vom 28.03.2024) vorliegt.

### Patientenpopulation

Insgesamt wurden 370 Patienten mit einem Durchschnittsalter von 69,4 Jahren (min. 50; max. 92) und einem Body-Mass-Index (BMI) von 24,9 kg/m^2^ (min. 16,2; max. 38,9) untersucht. Davon waren 53 Männer (Durchschnittsalter 68,7 Jahre, min. 51; max. 85; BMI 27,1 kg/m^2^, min. 19,7; max. 38,9) und 317 Frauen (Durchschnittsalter 69,4 Jahre, min. 50; max. 92; BMI 24,5 kg/m^2^, min. 16,2; max. 35,8); (Tab. 1). Die Untersuchung erfolgte zur Abklärung des Vorliegens einer Osteoporose. Die Zuweisungen erfolgten aus den Ambulanzen für Neurochirurgie, Orthopädie und Traumatologie, Geriatrie sowie Gynäkologie.

**Tab. 1:** Patientenbeschreibung nach Geschlecht, Alter, BMI und Anzahl der hüftgelenksnahen Frakturen sowie zusätzliche Spezifizierung der Frakturen. Das Durchschnittsalter der Frauen mit einer Fraktur liegt annähend 6 Jahren höher als das der Männer, des Weiteren finden sich bei den Frauen um ca. 13 % mehr sub-/pertrochantäre Frakturen als Schenkelhalsfrakturen, die Unterschiede sind mit *p* < 0,05 signifikant.

	Patienten (*n* = 370)	Männer (*n* = 53)	Frauen (*n* = 317)
⌀-Alter (in Jahren)	69,4 (min. 50; max. 92)	68,7 (min. 51; max. 85)	69,5 (min. 50; max. 92)
BMI (kg/m^2^)	24,9 (min. 16,2; max. 38,9)	27,1 (min. 19,7; max. 38,9)	24,5 (min. 16,2; max. 35,8)
Patienten mit einer Hüftfraktur *n* und (%)	58 (15,7 %)	5 (9,4 %)	53 (16,7 %)
Frakturtypen Schenkelhals sub-/pertrochantär *n* und (%)	28 von 58 (48,3 %)	4 von 5 (80 %)	23 von 53 (43,4 %)
	30 von 58 (51,7 %)	1 von 5 (20 %)	30 von 53 (56,6 %)
⌀-Alter der Patienten mit einer Fraktur (in Jahren)	75,9 (min. 52; max. 91)	70,2 (min. 59; max. 85)	76,5 (min. 52; max. 91)

*BMI* Body-Mass-Index

### Knochendichteauswertung

**Abb. 1:**
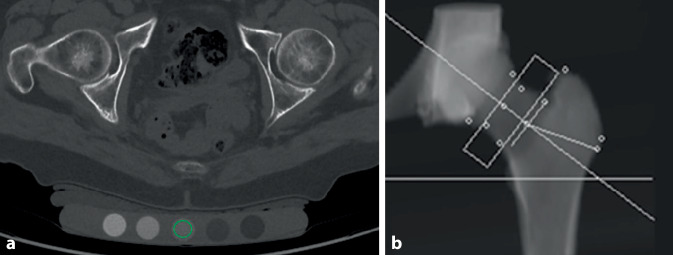
CTXA („computed tomography X‑ray absorptiometry“): Planung der trabekulären Bestimmung des Knochenmineralgehalt im CT-Schnittbild (**a, b**). Im Bild *links* *unten* Abbildung des mit erfassten Phantoms. Die nachträglich händisch eingelegte kreisrunde „region of interest“ (*grün*) in das mittlere Röhrchen zeigt eine Dichte von 50,91 HU, dieses passt zu der von der Industrie angegebenen Solldichte von 50 HU mit einer SD von ca. 4 HU (**a**). Der Wert ermöglicht approximativ eine Abschätzung einer möglichen Scannerschwankung und dient der Adjustierung

Es wurde der KMG der Hüfte in mg/cm^2^ mittels CTXA (GE-Revolution EVO/64 Zeilen CT mit der Mindways Software, CTXA-Hüfte, die den volumetrischen CT-Datensatz nutzt, Austin, TX, USA) ermittelt (Abb. 1), wobei auch die entsprechenden T‑Score-Werte dokumentiert wurden. Beide Hüften wurden ausgewertet, bei Vorliegen einer Hüft-TEP oder Fraktur wurde ausschließlich die gesunde Seite zur Berechnung des Mittelwerts herangezogen.

Patienten mit einem Alter < 50 Jahre, mit einem Malignom oder Zustand nach einem Hochrasanztrauma wurden von der Studie ausgeschlossen.

**Abb. 2:**
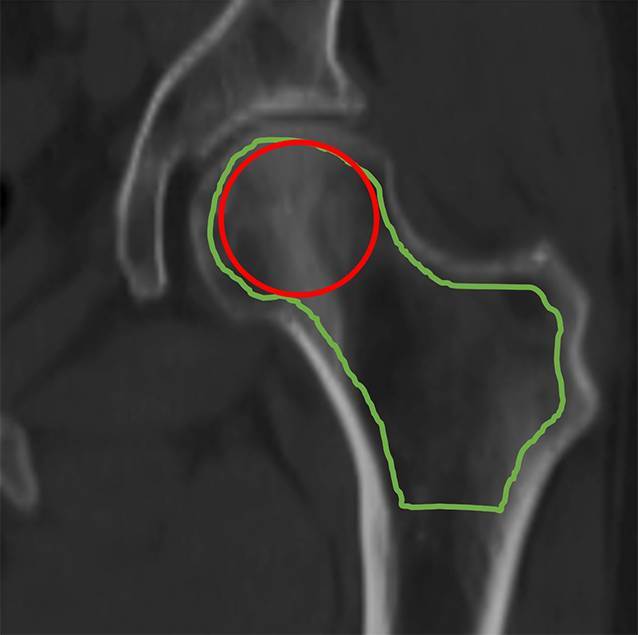
„Region of interest“ (ROI) im prox. Femur: kreisrunde ROI im Caput femoris (*rote Markierung*) sowie irreguläre Flächen-ROI (*grüne Markierung*) über die gesamte prox. Femurregion im koronar reformierten CT-Schnittbild

Im größten koronaren CT-Schnittbild wurde anschließend manuell mittels einer kreisrunden ROI im Caput femoris sowie einer irregulären Flächen-ROI über die gesamte prox. Femurregion die trabekuläre Dichte in HU bestimmt (Abb. 2). Der Abstand zur Kortikalis wurde mit etwa 1 mm festgelegt. Alle Untersuchungen wurden mit einer CT-Röhrenspannung von 120 kV durchgeführt. Die HU-Messungen erfolgten bei einer Schichtdicke von 2 mm und einer Fenstereinstellung von C = 400/W = 1600. Dieses Konzept wurde bereits in Vorgängerstudien erfolgreich angewandt und modifiziert übernommen [2, 49]. Die HU-Werte wurden anhand eines Referenzphantoms adjustiert.

### Frakturdetektion

Bei klinischem Verdacht wurden mögliche Hüftfrakturen auf konventionellen Röntgenaufnahmen sowie einer Becken-CT mit multiplanaren Reformationsaufnahmen erfasst. Die Frakturen wurden in Schenkelhals- und sub-/pertrochantäre Frakturen unterteilt.

### Statistik

Die erhobenen Daten wurden mit dem statistischen Softwarepaket SPSS, Version 23.0 (SPSS Inc., Armonk, NY, USA) analysiert. Quantitative Merkmale wurden bei parametrischen Tests als Mittelwert (M), Standardabweichung (SD) und Anzahl (*n*) der verfügbaren Beobachtungen dargestellt (M ± SD). Bei nichtparametrischen Tests erfolgte die Darstellung als Median mit erstem und drittem Quartil (Q1–Q3).

Zur Beschreibung des linearen Zusammenhangs zwischen zwei Variablen wurde der Pearson-Korrelationskoeffizient (r) berechnet. Mithilfe einer Regressionsanalyse wurden der CTXA-Wert (X_qHüfte_) unter Verwendung einer verallgemeinerten Schätzungsgleichung bestimmt. Zusätzlich erfolgte ein Vergleich der Korrelationskoeffizienten von HU-Werten mit den CTXA-Werten.

Mittels einer ROC-Analyse wurde die Vorhersagekraft für Osteoporose und Frakturen anhand der HU-Werte des Caput femoris und der gesamten Femurregion (Daten aus der irregulären Flächen-ROI) ermittelt. Um die AUC-Werte der beiden ROI-basierter Modelle zu vergleichen, wurde der DeLong-Test angewendet, um festzustellen, ob Unterschiede in der diagnostischen Leistung statistisch signifikant waren. Für den Vergleich der Patientengruppen – ohne und mit einer Hüftfraktur wurde der t‑Test zusätzlich herangezogen. Alle *p*-Werte basieren auf zweiseitigen statistischen Tests; grundsätzlich wird *p* < 0,05 als signifikant angesehen. Die Effektstärken nach Cohen wurden berechnet, wobei Werte < 0,5 als klein, zwischen 0,5 und 0,8 als mittel und > 0,8 als groß klassifiziert wurden.

## Ergebnisse

**Abb. 3:**
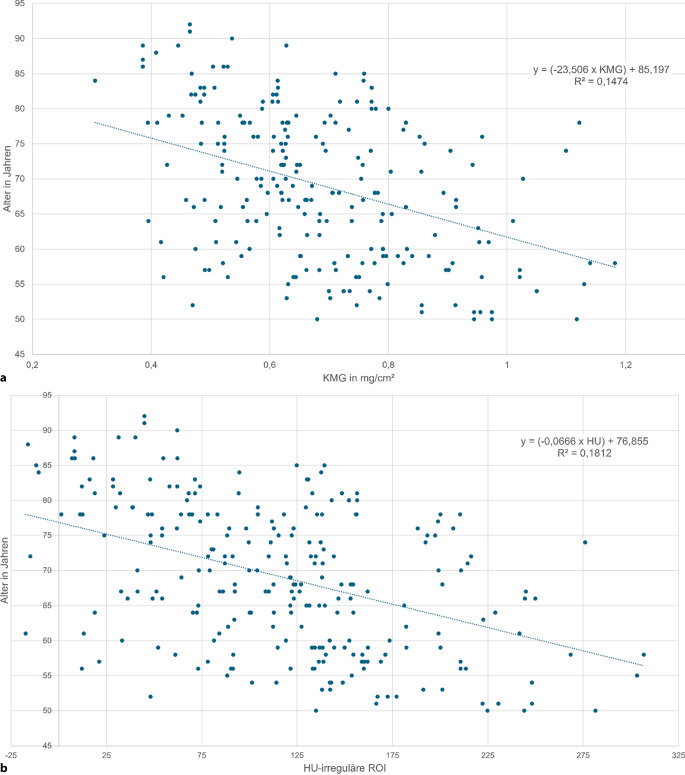
KMG(Knochenmineralgehalt)- (**a**) und HU(Hounsfield-Einheit)-Werte (**b**) der prox. Femurregion vs. Patientenalter: Es zeigt sich eine signifikante Abnahme von KMG- und HU-Werten mit zunehmendem Alter; *ROI* „region of interest“

**Abb. 4:**
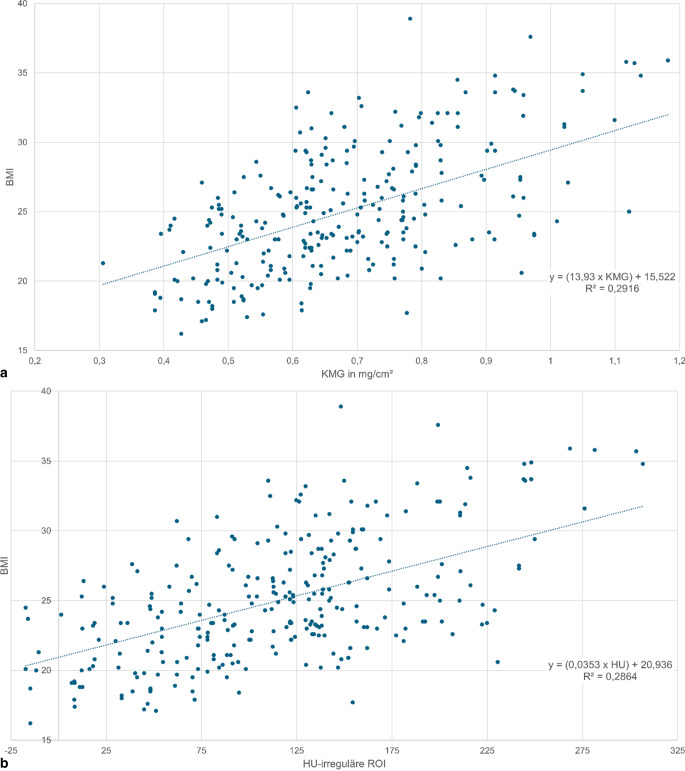
KMG(Knochenmineralgehalt)- (**a**) und HU(Hounsfield-Einheit)-Werte (**b**) der prox. Femurregion vs. BMI (Body-Mass-Index): Es zeigt sich eine signifikante Zunahme von KMG- und HU-Werten mit zunehmendem BMI; *ROI* „region of interest“

Bei Patienten mit zunehmendem Alter (Abb. 3a**,** b) und abnehmendem BMI (Abb. 4a, b) finden sich signifikant (*p* < 0,05) abnehmende KMG- und HU-Werte.

**Abb. 5:**
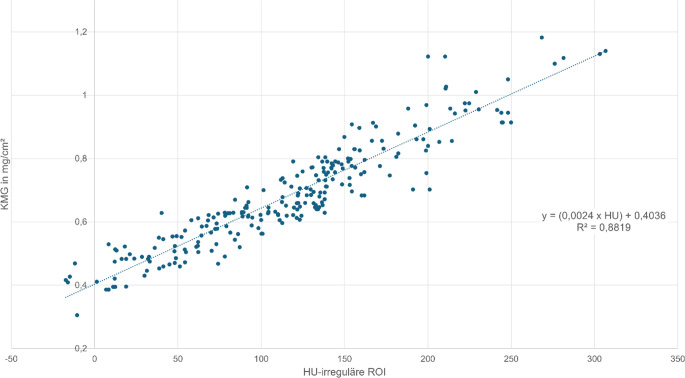
Bei einer Korrelation von R^2^ = 0,8819 (*p* < 0,001) lassen sich nach folgender Formel für die prox. Femurregion: Y = (0,0024 × HU) + 0,4036 quantitative Werte in mg/cm^2^ berechnen. Das 95 %-Konfidenzintervall für die Pearson-Korrelation zwischen KMG (Knochenmineralgehalt) und HU(Hounsfield-Einheit)-Wert der irregulären ROI („region of interest“) liegt bei r = 0,939 (95 % KI: 0,926 bis 0,950). Ein Wert von 96 HU ergibt 0,63 mg/cm^2^, dieses stellt die Grenze zur Osteoporose dar. Zur Berechnung der HU-Grenzwerte zur Osteoporose siehe Abb. 6

Für die gesamte Hüfte (Tab. 2 und 3) betrug der mediane KMG 0,646 (0,306–1,182) mg/cm^2^ und der mediane HU für die gesamte prox. Femurregion (Daten aus der irreguläre Flächen-ROI) 114,1 (−17,3–306,7). Bei einer Korrelation von R^2^ = 0,8819 (*p* < 0,001) lassen sich nach folgender Formel: X_ctxa_ = (0,0024 × HU) + 0,4036 aus den HU-Werten quantitative Werte in mg/cm^2^ errechnen (Abb. 5).

**Tab. 2:** KMG(Knochenmineralgehalt)- und T‑Score-Werte aus der CTXA („computed tomography X‑ray absorptiometry“).

KMG- und T‑Score-Werte der Hüfte					
	Patientenzahl (*n*)	Minimum	Maximum	Median	Mittelwert
KMG (mg/cm^2^)	370	0,306	1,182	0,646	0,673
T‑Score	370	−5,15	2,245	−2,38	−2,159

**Tab. 3:** HU(Hounsfield-Einheit)-Werte des Caput femoris und prox. Femurs.

HU-Werte					
	Patientenzahl (*n*)	Minimum	Maximum	Median	Mittelwert
HU Caput femoris	370	53,6	453,2	271,1	266,19
HU prox. Femur	370	−17,3	306,7	114,1	112,35

**Abb. 6:**
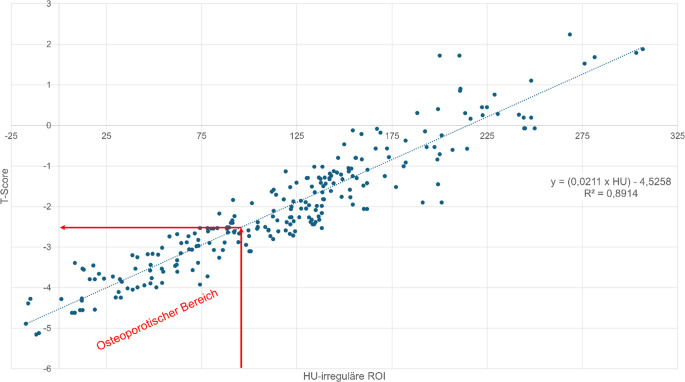
Korrelation HU (Hounsfield-Einheit) vs. T‑Score für die prox. Femurregion. Bei einer Korrelation von R^2^ = 0,8914 (*p* < 0,001) lassen sich nach folgender Formel für die prox. Femurregion: Y = (0,0211 × HU) −4,5258 T‑Score-Werte berechnen. Das 95 %-Konfidenzintervall für die Pearson-Korrelation zwischen dem T‑Score und den HU-Wert der irregulären ROI („region of interest“) liegt bei r = 0,944 (95 % KI: 0,932–0,954). Ein Wert von 96 HU ergibt einen T‑Score von −2,5, dieses stellt die Grenze zur Osteoporose dar. Die zuverlässigste Bestimmung der Osteoporoseschwelle gelingt mit der HU-Messung über die irreguläre Flächen-ROI im prox. Femur, mit einer Richtig-positiv-Rate von 97 %, hierzu siehe Abb. 10

Der mediane T‑Wert (Tab. 2) betrug −2,38 (−5,15 bis –2,245). Bei einer Korrelation von R^2^ = 0,8914 (*p* < 0,001) lassen sich nach folgender Formel X_tgesamt-irreguläre-Flächen-ROI_ = (0,0211 × HU) −4,5258 entsprechende T‑Werte aus den HU-Werten berechnen (Abb. 6). Hierbei entsprechen 96 HU einem T‑Wert von −2,5.

**Abb. 7:**
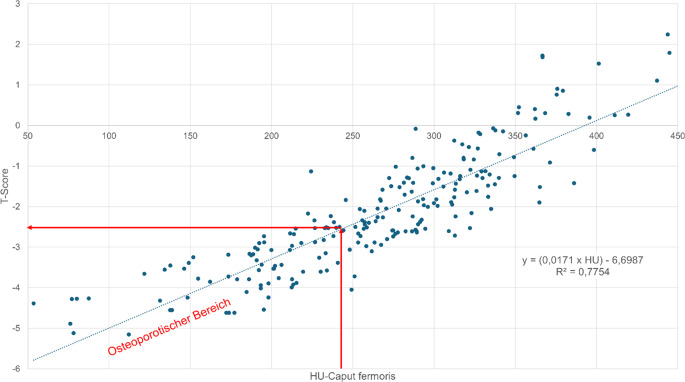
Korrelation HU (Hounsfield-Einheit) vs. T‑Score für das Caput femoris. Bei einer Korrelation von R^2^ = 0,7754 (*p* < 0,001) lassen sich nach folgender Formel für das Caput femoris: Y = (0,0171 × HU) −6,6987 T‑Score Werte berechnen. Das 95 %-Konfidenzintervall für die Pearson-Korrelation zwischen T‑Score und HU-Wert des Femurkopfes liegt bei r = 0,881 (95 % KI: 0,855–0,902). Ein Wert von 245,54 HU ergibt einen T‑Score von −2,5, dieses stellt die Grenze zur Osteoporose dar

Bei einer Korrelation von R^2^ = 0,7754 (*p* < 0,001) für das Caput femoris lassen sich nach folgender Formel X_tCaput femoris_ = (0,0171 × HU) −6,6987 entsprechende T‑Werte berechnen (Abb. 7). Hierbei entsprechen 245,54 HU einem T‑Wert von −2,5.

Unter Berücksichtigung der T‑Score-Werte zeigt Tab. 4 die prozentuale Verteilung des Patientenkollektivs in Normal, Osteopenie und Osteoporose. In Tab. 1 sind die Hüftfrakturen zusammengefasst. Bei den Patienten mit Hüftfrakturen sind Frauen im Vergleich mit den Männern um ca. 6 Jahre älter. Zusätzlich zeigen Frauen mit einem Alter über 80 Jahre deutlich mehr sub-/pertrochantäre Frakturen.

**Tab. 4:** Basierend auf dem T‑Score aus der CTXA („computed tomography X‑ray absorptiometry“) zeigt die Tabelle den prozentualen Anteil der Patienten mit normwertigem KMG (Knochenmineralgehalt), Osteopenie und Osteoporose. Für die Gruppierungen wurden die Grenzwerte des T‑Scores verwendet, wie sie in der DVO-Leitlinie Osteoporose festgelegt sind [21].

Verteilung der Patienten	
Normaler KMG (T-Score > −1)	62 von 370 (16,8 %)
Osteopenie (T-Score −1 bis −2,5)	138 von 370 (37,3 %)
Osteoporose (T-Score < −2,5)	170 von 370 (45,9 %)

**Abb. 8:**
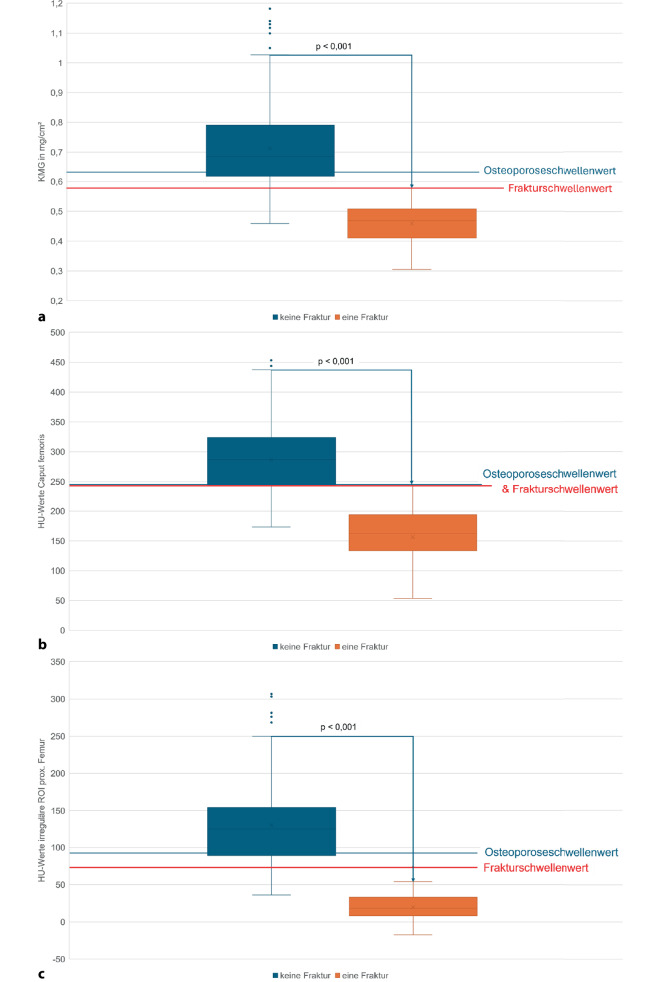
**a** KMG(Knochenmineralgehalt)-, **b** HU(Hounsfield-Einheit)-Werte Caput femoris und **c** HU-Werte prox. Femur vs. Frakturen Hüfte. Oberhalb der Osteoporoseschwellenwerte finden sich keine Hüftfrakturen. Mit allen Messungen ist eine signifikante Trennung zwischen den Patienten ohne und mit einer Fraktur möglich, die zuverlässigste Bestimmung der Frakturschwelle gelingt jedoch mit der HU-Messung über die irreguläre Flächen-ROI („region of interest“) im prox. Femur, mit einer Richtig-positiv-Rate von 99 %, hierzu siehe Abb. 9

**Abb. 9:**
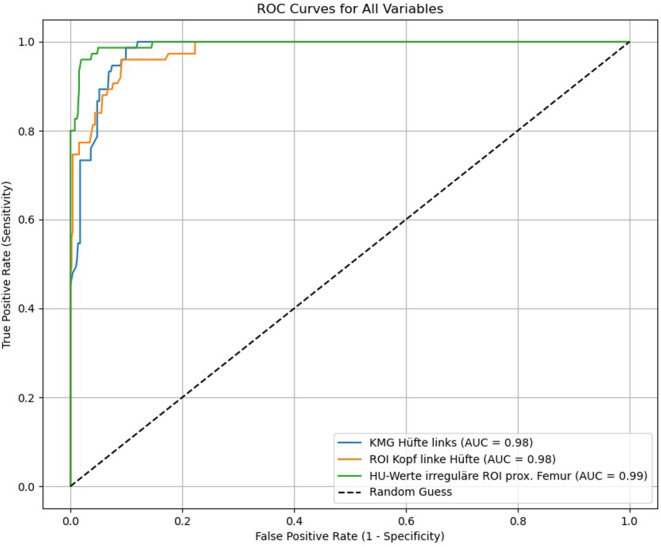
ROC („receiver operating characteristic“) KMG(Knochenmineralgehalt)-, HU(Hounsfield-Einheit)-Werte Caput femoris und HU-Werte prox. Femur vs. Frakturrisiko der Hüfte. Hierbei zeigt die irreguläre Flächen-ROI („region of interest“) des prox. Femurs mit einer AUC („area under the curve“) = 0,99 die höchste Frakturprädiktion. Gefolgt von der kreisrunden ROI im Caput femoris mit einer AUC = 0,98 und der KMG-Messung ebenfalls mit einer AUC = 0,98

Mittels CTXA (KMG-Bestimmung) und der trabekulären Dichtemessungen in HU lassen sich Patienten ohne und mit einer Hüftfraktur signifikant diskriminieren (Abb. 8a, b, c), wobei die Dichtebestimmung über die Flächen-ROI des prox. Femurs mit 99 % in der AUC die höchste Vorhersagekraft aufweist (Abb. 9).

**Abb. 10:**
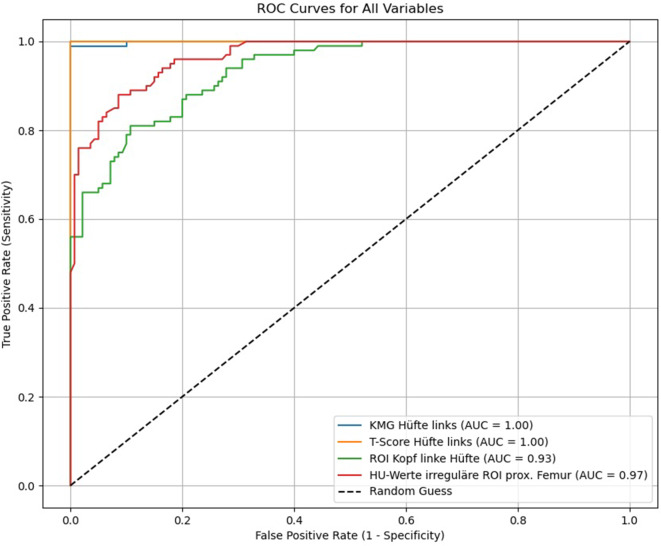
ROC („receiver operating characteristic“) KMG(Knochenmineralgehalt)-, HU(Hounsfield-Einheit)-Werte Caput femoris und HU-Werte prox. Femur vs. Osteoporoseschwellenwert. Mittels ROC-Kurvenanalyse lässt sich zeigen, dass HU-Werte der gesamten prox. Femurregion in der Osteoporoseabschätzung eine hohe Übereinstimmung mit den KMG- (mg/cm^2^) und den T‑Werten aus der CTXA („computed tomography X‑ray absorptiometry“) aufweisen. Hierbei zeigt die irreguläre Flächen-ROI („region of interest“) des prox. Femurs mit einer AUC = 0,97 die höhere Osteoporosevorhersagekraft gegenüber der kreisrunden ROI im Caput femoris mit einer AUC = 0,93

Mittels ROC-Kurvenanalyse lässt sich zeigen, dass HU-Werte der gesamten prox. Femurregion (Daten aus der irreguläre Flächen-ROI) in der Osteoporosevorhersagekraft eine hohe Übereinstimmung (AUC = 0,97) mit den KMG- (mg/cm^2^; Sensitivität = 0,92; Spezifität = 0,93) und den T‑Werten (Sensitivität = 0,92; Spezifität = 0,93) bei einem Osteoporoseschwellenwert von 96 HU aufweisen und sich mit einem *p* = 0,398 kein signifikanter Unterschied findet. Hierfür ergibt sich eine Effektstärke von 0,91. Des Weiteren lässt sich zeigen, dass HU-Werte des Caput femoris (Daten aus der kreisrunden ROI) in der Osteoporosevorhersagekraft eine Übereinstimmung (AUC = 0,93) mit den KMG- (mg/cm^2^; Sensitivität = 0,89; Spezifität = 0,93) und den T‑Werten (Sensitivität = 0,92; Spezifität = 0,93) bei einem Osteoporoseschwellenwert von 245,54 HU aufweisen und sich mit einem *p* = 0,412 nicht unterscheiden. Hierfür ergibt sich eine Effektstärke von 0,88 (Abb. 10).

## Diskussion

Im Vergleich mit der DEXA-Hüfte ist die trabekuläre Dichtebestimmung in HU im koronaren CT-Schnittbild des Femurs frei von degenerativ bedingten Überlagerungen und ohne Aufsummierung der Kortikalis. Durch eine individuell eingebrachte, möglichst bis kurz vor der Kortikalis endenden ROI ist es möglich, die trabekuläre Dichte im Caput femoris und des prox. Femurs selektiv zu erfassen (Abb. 2). Der KMG und die trabekuläre Gesamtdichte des prox. Femurs in HU zeigt eine signifikante Abnahme mit zunehmendem Patientenalter (Abb. 3a, b) und abnehmendem BMI (Abb. 4a, b), dieses untermauert ein hohes Patientenalter und niedrigen BMI als Risikofaktoren für Osteoporose [4]. Für das Auftreten von Hüftfrakturen ist ebenfalls das höhere Alter ein Risikofaktor, wobei sich in unserer Studie bei Frauen mit einem Durchschnittsalter von 76,5 Jahren vermehrt sub-/pertrochantäre Frakturen finden (Tab. 1). Hierzu passen auch die Daten von Crilly et al. [13], wobei der relative Anteil von intertrochantären Frakturen bei Frauen von 35 % in der Altersgruppe (55–59 Jahre) auf 51 % in der älteren Gruppe (84+ Jahre; *p* < 0,0001) stieg.

Mittels ROC-Kurvenanalyse lässt sich zeigen, dass HU-Werte der gesamten prox. Femurregion (Daten aus der irregulären Flächen-ROI) in der Osteoporosevorhersagekraft eine hohe Übereinstimmung mit den KMG- (mg/cm^2^; Sensitivität = 0,92; Spezifität = 0,92) und den T‑Werten (Sensitivität = 0,92; Spezifität = 0,93) bei einem Osteoporoseschwellenwert von 96 HU aufweisen und sich mit einem *p* = 0,395 kein signifikanter Unterschied findet. Die Bestimmung der HU-Werte über die gesamte prox. Femurregion zeigt im Vergleich mit den Dichtewerten für das Caput femoris die höchste Aussagekraft (AUC = 0,97) zur Vorhersage einer Osteoporose (Abb. 10), dieses findet sich auch in gleicher Weise in der Vorgängerstudie von Andresen et al. [2]. Zhao et al. [49] finden mit einer hohen Vorhersagekraft einen Osteoporosegrenzwert für den linken prox. Femur von 192,23 HU und für den rechten prox. Femur von 188,71 HU, die Werte überschreiten unsere Werte bei weitem, da hier die Kortikalis mitgemessen wurde. Trotzdem zeigt die gesamte prox. Femurregion mit einem r = 0,826 einen positiven Zusammenhang zwischen den HU- und KMG-Werten, welches bei uns mit einem R^2^ = 0,8819 noch deutlicher ausfällt.

Für die trabekuläre Dichte im Caput femoris findet sich in unserer Untersuchung bei 245,54 HU, diese entspricht einem T‑Score von −2,5, die Grenze zur Osteoporose (Abb. 7). Lee et al. [29] finden einen Grenzwert für Osteoporose von 296,15 HU, die Werte sind deutlich höher als unsere Werte, dieses ist bedingt durch eine kleinere, zentral in die höchste trabekulären Dichte des Caput femoris gelegte ROI. Im Vergleich zur DXA-Messung finden auch Kilinc et al. [28] eine starke Korrelation mit den HU-Werten des Caput femoris, wobei der Osteoporosegrenzwert der linken Hüfte bei 198,24 HU und der rechten Hüfte bei 204,65 HU liegt. Hier kommen die Autoren im Vergleich zu unseren jetzigen Daten und der Vorgängerstudie [2] zu deutlich niedrigeren Osteoporosegrenzwerten, dieses ist durch eine unterschiedlich große ROI, mit mehr Abstand zur Kortikalis in unserer Methode zu begründen. Fan et al. [17] konnten zeigen, dass bei HU-Werten der trabekulären Dichte des Caput femoris von < 124,85, Werte die deutlich im osteoporotischen Bereich liegen, das Risiko für ein Implantatversagen nach Versorgung einer intertrochantären Fraktur mit einem intramedullären Nagel signifikant erhöht ist. Auch mittels ROI im Collum femoris ist eine Dichtebestimmung zur Osteoporoseabschätzung ebenfalls gut möglich [2, 23, 31, 49], eine beginnende Demineralisation und Strukturrarefizierung ist hier sogar sensitiver als im Caput femoris zu erfassen, dieses lässt sich mittels Flächen-ROI über das prox. Femur gut ausgleichen und genauer darstellen [2]. Da die Grenze zur trabekulären Struktur in Richtung Caput femoris und der intertrochantären Region nicht einfach eingehalten werden kann, wurde die Dichtebestimmung mittels ROI im Colum femoris bei uns nicht weiter berücksichtig, ähnliches gilt für die ROI-Platzierung in der pertrochantären Region.

Neben der Osteoporoseabschätzung lässt sich mit der Dichtebestimmung in HU auch signifikant (*p* < 0,001) zwischen Patienten ohne und mit einer Fraktur diskriminieren (Abb. 8b, c). Für das Caput femoris liegt der Frakturschwellenwert bei 245,54 HU und für das prox. Femur bei 75 HU. Zur Vorhersagekraft einer Hüftfraktur ist die Richtig-positiv-Rate mit 99 % für die Flächen-ROI des prox. Femurs am höchsten, im Vergleich zur Dichtebestimmung mittels ROI im Caput femoris mit 98 % und der KMG-Bestimmung mittels CTXA mit 98 %. Narayanan et al. [33] finden in der Dichtemessung im Caput femoris eine mittlere Dichte von 279,7 HU bei Patienten mit einer Fraktur und eine mittlere Dichte von 410,6 HU in der Kontrollgruppe, der Unterschied ist wie bei uns signifikant (Abb. 8b), wobei die mittleren Dichtewerte bei unterschiedlich großer ROI selbst nicht vergleichbar sind.

Insgesamt werden Unterschiede der angegebenen Grenzwerte durch nicht vergleichbare Patientenkollektive [1] und unterschiedliche technische Parameter, wie kV-Werte der CT-Röhrenspannung, Fenstereinstellungen, Schichtdicken und ROI-Größen sowie Formen mit nicht definiten Grenzen zur Kortikalis, Zentrierungen der ROI sowie CT-Schnittebenen hervorgerufen [2, 8, 17, 23, 28, 29, 31, 33, 42, 49]. Automatische Konturfindungsprogramme, welche leicht zu implementieren sind, könnten die Messungen weiter optimieren. Für eine Vergleichbarkeit von Literaturdaten ist hier eine Standardisierung dringend notwendig. Wenn dieses berücksichtigt wird, ist eine Osteoporoseabschätzung aus vorhandenen nativen CT-Schnittbilder ohne weite Untersuchungen gut möglich. Als Surrogatparameter lässt sich dieses dann für eine Frakturrisikobewertung [9] und gegebenenfalls medikamentöse Therapieplanung [40] heranziehen.

Generell bekommt die trabekuläre Dichtebestimmung in HU zur Abschätzung einer Osteoporose, ossären Strukturrarefizierung und einer Frakturrisikobewertung in CT-Schnittbildern eine immer größere Bedeutung im klinischen Alltag, wobei sich valide Ergebnisse für die Hüftregion [2, 8, 9, 17, 23, 28, 29, 31, 33, 42, 44–47, 49] und am Achsenskelett [1, 3, 5, 11, 30, 34, 36, 38] finden. Gezielte Dichtemessungen im Dens axis [37], der Halswirbelsäule [18] und dem prox. Humerus [32] erscheinen für eine lokale Strukturanalyse und Frakturrisikobewertung ebenfalls möglich und hilfreich.

Weitere Forschung ist erforderlich, um HU-basierte Schwellenwerte für verschiedene Patientenpopulationen, Altersgruppen, Geschlechter und anatomische Regionen zu optimieren und automatisierte Methoden zur standardisierten ROI-Auswahl zu ermöglichen. Die Einbeziehung HU-basierter Knochendichtemessungen in Modelle zur Frakturrisikovorhersage könnte dazu beitragen, individualisierte Behandlungspläne zu optimieren und osteoporotischen Frakturen bei Hochrisikopatienten vorzubeugen. Darüber hinaus erscheint die Datenerfassung und standardisierte Auswertung von HU-Werten von verschiedenen CT-Scannern mit unterschiedlichen Untersuchungsprotokollen und heterogenen Untersuchungskollektiven durch den Einsatz KI-basierter Bildanalyse, einschließlich der Implementierung automatisierter Segmentierungstechniken auf der Basis von Algorithmen des maschinellen Lernens zur Reduzierung der Variabilität zwischen und innerhalb der Untersucher, vielversprechend, um die Reproduzierbarkeit und Präzision weiter zu erhöhen. Somit wird die klinische Anwendbarkeit der HU-basierten Osteoporosediagnostik und Frakturrisikobestimmung in größerem Maßstab ermöglicht, was Klinikern durch verfügbare Open-Source-Tools bereits jetzt möglich ist [41].

### Limitationen und Stärken

Es handelt sich um eine retrospektive Studie. Die Patientenkohorte basiert nicht auf einer zufälligen Auswahl der Allgemeinbevölkerung, sondern auf Überweisungen spezialisierter Ambulanzen. Dies könnte zu einem Selektionsbias führen und die Generalisierbarkeit der Ergebnisse einschränken. Demgegenüber weist das Patientenkollektiv eine große Bandbreite an Alter und BMI auf, die einem breiten Bevölkerungsanteil entsprechen dürfte und durch die zusätzliche Zahl von 370 Patienten für die Korrelationsberechnungen eine hohe Aussagekraft ermöglicht.

Die manuell eingefügten ROI-Größen und -Platzierungen unterliegen einer gewissen Variabilität. Die HU-Werte wurden mit den CTXA-Daten und nicht mit dem Goldstandard DXA verglichen.

### Schlussfolgerung

Diese Studie zeigt, dass Messungen der trabekulären Knochendichte in HU aus nativen CT-Schnittbildern eine zuverlässige Methode zur Beurteilung von Osteoporose und Frakturrisiko des prox. Femurs darstellen.

Die starken Korrelationen zwischen HU-Werten und KMG-Messungen mittels CTXA-Hüfte legen nahe, dass HU-basierte Messungen als wirksames Surrogat für die Diagnose von Osteoporose dienen können. Bei Folgeuntersuchungen erscheint eine Verlaufsbeurteilung möglich. Unsere Ergebnisse zeigen, dass ein Schwellenwert von 96 HU über den gesamten prox. Femurbereich und 245,54 HU für das Caput femoris, einem T‑Wert von −2,5 entspricht, dem Standarddiagnoseschwellenwert für Osteoporose. Oberhalb dieser Osteoporoseschwellenwerte finden sich keine Hüftfrakturen. Hierbei zeigt jeweils die Flächen-ROI des prox. Femurs die präziseste Korrelation, mit einer Richtig-positiv-Rate von 99 %.

Angesichts der weit verbreiteten Verfügbarkeit einer CT-Bildgebung könnte die Integration der HU-basierten Osteoporosediagnostik in die klinische Praxis die Früherkennung und Interventionsstrategien verbessern und letztlich das Outcome der Patienten optimieren.

## Fazit für die Praxis

- Unter Berücksichtigung der gewonnenen Ergebnisse erscheint eine opportunistische Auswertung mittels Hounsfield-Einheiten im nativen CT-Schnittbild zur Abschätzung einer Osteopenie/Osteoporose und Bestimmung des Risikos für osteoporotische Hüftfrakturen möglich.
- Bei bereits vorhandenen CT-Untersuchungen könnten diese genutzt werden, es entfallen weitere Untersuchungszeiten, eine erneute Strahlenexposition und zusätzliche Kosten.

## Data Availability

Die erhobenen Datensätze können auf begründete Anfrage in anonymisierter Form beim korrespondierenden Autor angefordert werden. Die Daten befinden sich auf einem Datenspeicher am Westküstenklinikum Heide, Deutschland.

## Abkürzungen

AUC: „Area under the curve“
BMD: „Bone mineral density“
BMI: Body-Mass-Index
CTXA: „Computed tomography X‑ray absorptiometry“
DEXA: „Dual-energy X‑ray absorptiometry“
GE: General Electric
HU: Hounsfield-Einheit
Hüft-TEP: Hüfttotalendoprothese
KMG: Knochenmineralgehalt
M: „Mean value“/Mittelwert
n: „Number“/Anzahl
prox.: Proximal
Q1–Q3: Erstes und drittes Quartil
ROC: „Receiver operating characteristic“
ROI: „Region of interest“
R^2^: Gütemaß der linearen Regression
SD: „Standard deviation“/Standardabweichung
T‑Score: Der T‑Score ist ein Maß, das die Knochendichte eines Patienten mit der von gesunden jungen Erwachsenen vergleicht
